# Development of a claims-based risk-scoring model to predict emergency department visits in older patients receiving anti-neoplastic therapy

**DOI:** 10.1038/s41598-024-51981-0

**Published:** 2024-01-17

**Authors:** Yewon Suh, Jonghyun Jeong, Soh Mee Park, Kyu‑Nam Heo, Mee Yeon Lee, Young-Mi Ah, Jin Won Kim, Kwang-il Kim, Ju-Yeun Lee

**Affiliations:** 1 College of Pharmacy and Research Institute of Pharmaceutical Sciences, Seoul National University, 1 Gwanak-ro, Gwanak-gu, Seoul, 08826 Republic of Korea; 2 Department of Pharmacy, Seoul National University Bundang Hospital, Seongnam, Gyeonggi-do Republic of Korea; 3 College of Pharmacy, Yeungnam University, Gyeongsan, Gyeongbuk Republic of Korea; 4 Division of Hematology and Medical Oncology, Department of Internal Medicine, Seoul National University Bundang Hospital, Seongnam, Gyeonggi-do Republic of Korea; 5 Department of Internal Medicine, Seoul National University College of Medicine, Seoul, Republic of Korea; 6 Division of Geriatrics, Department of Internal Medicine, Seoul National University Bundang Hospital, Seongnam, Gyeonggi-do Republic of Korea

**Keywords:** Geriatrics, Chemotherapy, Risk factors, Palliative care

## Abstract

This study developed and validated a risk-scoring model, with a particular emphasis on medication-related factors, to predict emergency department (ED) visits among older Korean adults (aged 65 and older) undergoing anti-neoplastic therapy. Utilizing national claims data, we constructed two cohorts: the development cohort (2016–2018) with 34,642 patients and validation cohort (2019) with 10,902 patients. The model included a comprehensive set of predictors: demographics, cancer type, comorbid conditions, ED visit history, and medication use variables. We employed the least absolute shrinkage and selection operator (LASSO) regression to refine and select the most relevant predictors. Out of 120 predictor variables, 12 were integral to the final model, including seven related to medication use. The model demonstrated acceptable predictive performance in the validation cohort with a C-statistic of 0.76 (95% CI 0.74–0.77), indicating reasonable calibration. This risk-scoring model, after further clinical validation, has the potential to assist healthcare providers in the effective management and care of older patients receiving anti-neoplastic therapy.

## Introduction

In older patients with cancer, the complexity of medication regimens, combined with multiple comorbidities, significantly increase the risk of adverse clinical outcomes, including emergency department (ED) visits and hospitalizations^1,2^. A previous study showed that 14.3% of all ED visits were medication-related, with 76% being preventable^3^. Specifically, medication-related issues are a leading cause of ED visits among older adults, with anti-neoplastic agents accounting for 9.6% of the adverse drug events associated with ED visits in this population^4^. Furthermore, the issue of inappropriate polypharmacy in older patients on anti-neoplastic therapy, which further elevates the risk of ED visits, has been highlighted in previous studies^5,6^.

ED visits during cancer treatment adversely affect treatment efficacy, contribute significantly to the rising costs of cancer care^7^, and are associated with longer hospital stays, delayed treatment, and worse outcomes^8,9^. Therefore, preventing ED visits in patients with cancer is crucial for improved clinical outcomes, leading to the development of various prediction models for identifying high-risk patients.

Among the existing predictive models, the work of Brooks et al. is noteworthy^10,11^. They developed two models to predict hospitalization risks in cancer patients using American hospital data. Their first model focused on predicting chemotherapy-related hospitalization in patients starting palliative chemotherapy. However, this model’s scope in considering medication factors was limited to the type of chemotherapy used^10^. Their second model aimed to predict hospitalization within 30 days of starting chemotherapy, incorporating only non-medication variables like serum albumin concentration, and omitting a broader range of medication-related factors^11^. Other studies have developed models targeting specific cancer types^12–14^, limiting their wider applicability. Models by Grant RC et al. and Sutradhar R et al., targeted hospitalization or ED visits among patients undergoing chemotherapy using administrative databases, but they failed to include medication-related variables, a critical component of cancer care^15,16^.

This review of existing literature reveals a consistent trend: most predictive models for ED visits or hospitalizations in cancer patients either focus on entire adult population, disregarding the unique aspects of older populations, or fail to incorporate comprehensive medication-related variables. Additionally, there’s a notable lack of studies using large, population-based data to develop these predictive models.

Our study aimed to address this gap by developing and validating a risk-scoring model that incorporates a wide range of medication-related factors for older Korean patients undergoing anti-neoplastic therapy, using extensive population-based claims data. This approach not only fills a gap in current research but also capitalizes the advantages of national claims data, including automated data acquisition and broad applicability. Particularly, integrating this model with a national prospective Drug Utilization Review system^17^, will allow identifying high-risk populations without additional assessments.

By focusing on older patients and integrating detailed medication histories, our model aims to provide a more accurate and clinically relevant tool for predicting ED visits, thereby contributing to improved management and outcomes of this vulnerable population.

## Results

### Population characteristics

**Figure 1:**
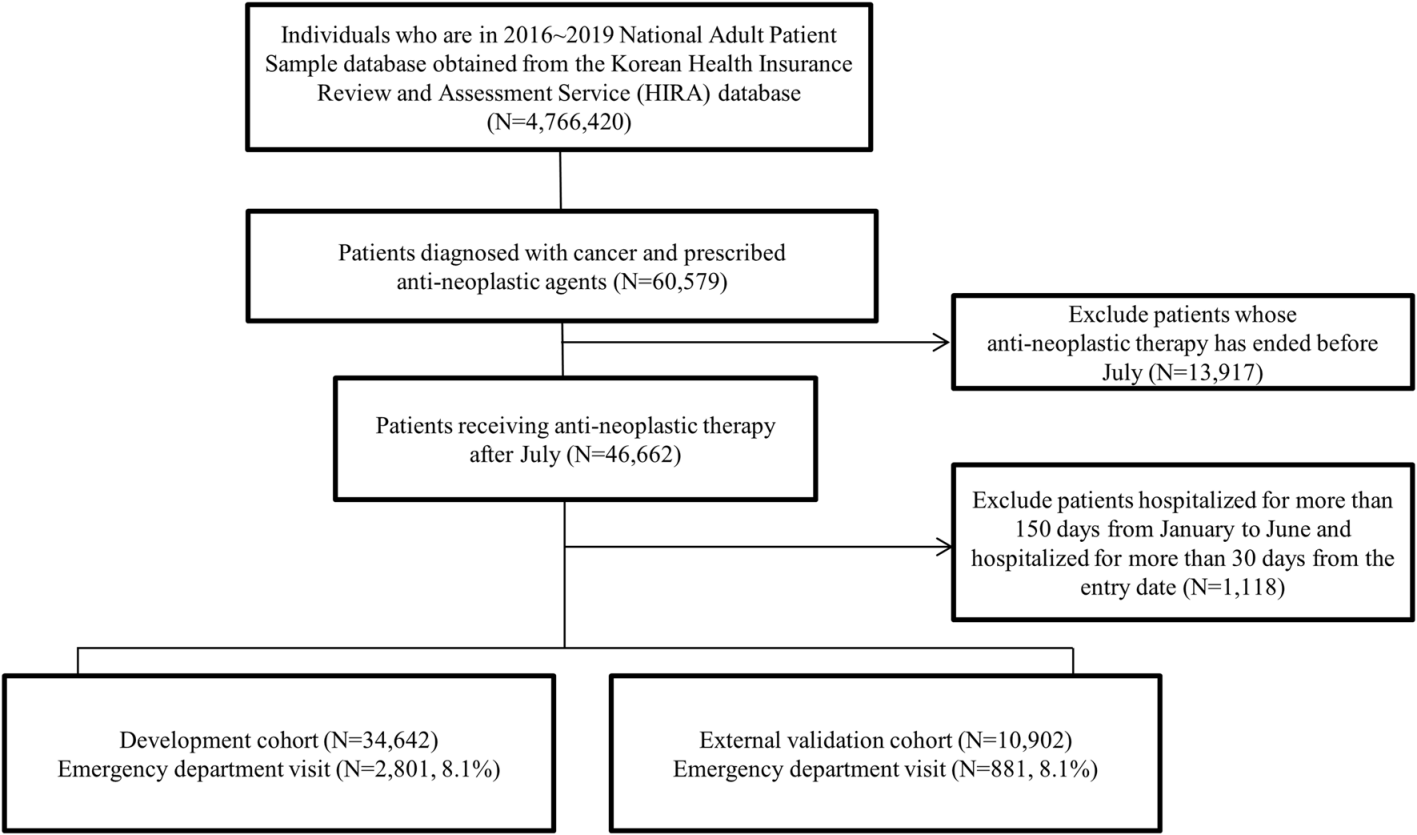
Flow chart depicting the methodology of patient selection.

We identified 60,579 patients diagnosed with cancer and prescribed anti-neoplastic agents. Among these, 46,662 patients were prescribed anti-neoplastic agents after July. After excluding patients hospitalized for more than 150 days from January to June and those hospitalized for more than 30 days from the entry date, 45,544 patients met the inclusion criteria. Among them, 34,642 patients were assigned to the development cohort and 10,902 patients to the external validation cohort. The number of patients who visited the ED within 30 days of entry date was 2801 (8.1%) in the development cohort and 881 (8.1%) in the external validation cohort (Fig. 1). The patients in both cohorts had similar characteristics and distributions of outcomes (Table 1).

**Table 1:** Baseline characteristics of study participants in the development and external validation cohorts.

Characteristics, N (%)	Development cohort	External validation cohort
	N = 34,642	N = 10,902
Age group		
65–74 years	19,636 (56.7)	6048 (55.5)
≥ 75 years	15,006 (43.3)	4854 (44.5)
Sex, male	21,603 (62.4)	6620 (60.7)
Insurance type		
Health insurance	31,796 (91.8)	9828 (90.1)
Medical aid and national meritorious service	2846 (8.2)	1074 (9.9)
A history of ED visits within 3 months	5086 (14.7)	1612 (14.8)
Cancer diagnosis		
Prostate cancer	10,354 (29.9)	3154 (28.9)
Breast cancer	6574 (19.0)	2180 (20.0)
Lung cancer	4308 (12.4)	1268 (11.6)
Colon cancer	3812 (11.0)	970 (8.9)
Comorbidities or prior condition		
Hypertension	22,671 (65.4)	7174 (65.8)
Diabetes mellitus	14,106 (40.7)	4510 (41.4)
Major bleeding	6618 (19.1)	1994 (18.3)
Atrial fibrillation	1759 (5.1)	597 (5.5)
Charlson comorbidity index score		
0–6	24,513 (70.8)	8391 (77.0)
> 6	10,129 (29.2)	2511 (23.0)
Initiation of anti-neoplastic therapy	11,564 (33.4)	3459 (31.7)
Type of anti-neoplastic agents		
Cytotoxic agent-based	16,573 (47.8)	5084 (46.6)
Chemotherapeutic drug interactions	6569 (19.0)	2112 (19.4)
Use of three or more CNS-active drugs^a^	2665 (7.7)	892 (8.2)
Regular use of opioids without laxatives	1280 (3.7)	399 (3.7)
Use of megestrol	1782 (5.1)	489 (4.5)
Use of 10 or more medications		
0–4	23,566 (68.0)	7630 (70.0)
5–9	3475 (10.0)	1022 (9.4)
≥ 10	7601 (21.9)	2250 (20.6)

*ED* emergency department, *CNS* central nervous system.

^a^CNS-active drugs: antiepileptics; antipsychotics; benzodiazepines; nonbenzodiazepine, benzodiazepine receptor agonist hypnotics; tricyclic antidepressants; selective serotonin reuptake inhibitors; serotonin-norepinephrine reuptake inhibitors; and opioids.

### Model development and validation

In the initial stage of model development, from the 120 predictors initially screened (Table S1), 59 candidate predictors were excluded due to a frequency of less than 1%. These included specific two cancer diagnoses, 16 general potentially inappropriate medications (PIMs), 15 geriatric drug-drug interactions (DDIs), and 26 disease-specific PIMs. Following this, we assessed multicollinearity among the remaining variables, but none were excluded as all had a variance inflation factor below 10. This led to 61 candidate predictors being subjected to the least absolute shrinkage and selection operator (LASSO) regression analysis. We selected a lambda value of λ = 0.00129, corresponding to log (λ) = − 2.9 after plotting the binomial deviance curve against log lambda (λ) and the coefficient profile against the log (λ) sequence (Fig. S1). This choice resulted in retaining 39 variables for the LASSO regression model. Further refinement, based on the regression coefficients and excluding variables with coefficients below 1 when multiplied by 100, narrowed this down to 12 final variables, forming the basis of predictive model. The final model included variables such as a history of ED visits within the past 3 months, a diagnosis of lung cancer, two specific comorbid conditions (atrial fibrillation and major bleeding), a Charlson comorbidity index score of 6 or higher, and seven medication-related factors. These factors are the initiation of anti-neoplastic therapy, anti-neoplastic therapy with cytotoxic agents, chemotherapeutic DDIs, use of three or more central nervous system (CNS)-active drugs, regular use of opioids without laxatives, use of megestrol, and the use of 10 or more medications. Each variable received a weighted score based on its coefficients in the LASSO regression model, as shown in Table 2: a history of ED visits within 3 months was assigned the highest weight of 13 points, followed by lung cancer, anti-neoplastic therapy with cytotoxic agents, use of three or more CNS-active drugs, and regular use of opioids without laxatives, each assigned 3 points. Two points were allocated for the initiation of anti-neoplastic therapy, chemotherapeutic drug interactions, and the use of megestrol. The risk scores ranged from 0 to 35 (Table 2).

**Table 2:** The risk scores and beta coefficients in the prediction model for emergency department visits in older patients receiving anti-neoplastic therapy.

Predictive factor	β Coefficient	Score
A history of ED visits within 3 months	0.1331	13
Cancer diagnosis		
Lung cancer	0.0258	3
Comorbidities or prior condition		
Atrial fibrillation	0.0117	1
Major bleeding	0.0119	1
Charlson comorbidity index score		
> 6	0.0109	1
Initiation of anti-neoplastic therapy	0.0249	2
Type of anti-neoplastic agents		
Cytotoxic agents-based	0.0295	3
Chemotherapeutic drug interactions	0.0157	2
Use of three or more CNS-active drugs^a^	0.0262	3
Regular use of opioids without laxatives	0.0307	3
Use of megestrol	0.0211	2
Use of 10 or more medications		
≥ 10	0.0133	1

*ED* emergency department, *CNS* central nervous system.

^a^CNS-active drugs: antiepileptics; antipsychotics; benzodiazepines; nonbenzodiazepine, benzodiazepine receptor agonist hypnotics; tricyclic antidepressants; selective serotonin reuptake inhibitors; serotonin-norepinephrine reuptake inhibitors; and opioids.

**Figure 2:**
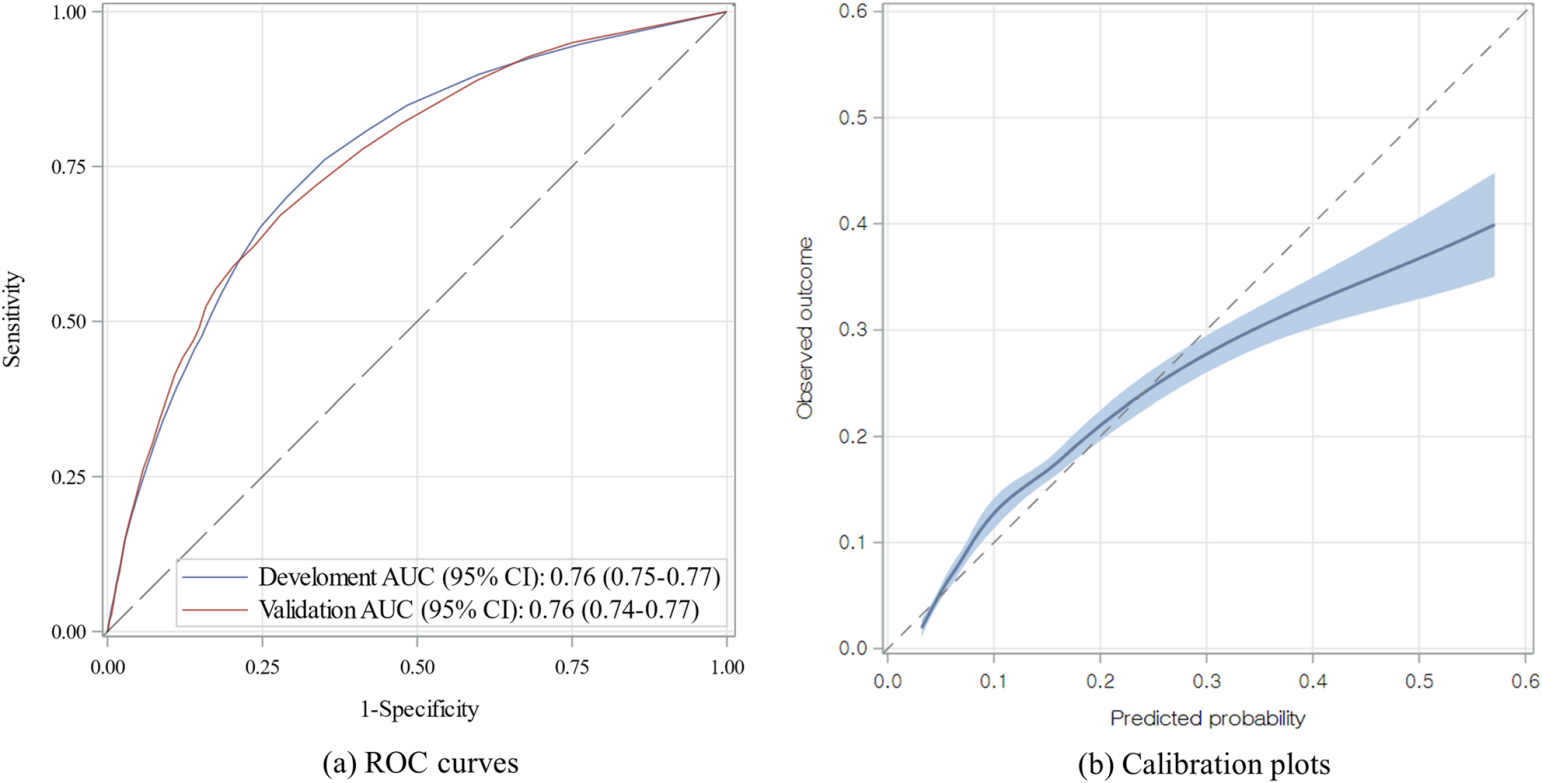
(**a**) Receiver operating characteristic (ROC) curves in the development and external validation cohorts. (**b**) Calibration plots in the external validation cohort.

For validation, the area under the receiver operating characteristic curve (AUROC) of the prediction model in both the development and external validation cohorts was 0.76 (95% confidence interval (CI) 0.75–0.77 and 0.74–0.77, respectively) (Fig. 2a). The calibration of the model was generally accurate, though a slight overestimation was observed for scores above 25(Fig. 2b and Table S2).

### Risk stratification

The cut-off value for high-risk classification was set at 7 points. Table 3 presents the stratification results for both the development and external validation cohorts. In the external validation cohort, 30.9% (3365 patients) were categorized as high-risk and 69.1% (7537 patients) as low-risk. Among these groups, 590 (17.5%) of the high-risk and 291 (3.9%) of the low-risk patients eventually visited the ED. The sensitivity and specificity of the model were determined to be 67.0% (95% CI 63.9–70.1%) and 72.3% (95% CI 71.4–73.2%), respectively. The top three cases predicted as high-risk by our model are detailed in Table S3.

**Table 3:** Accuracy of the risk-scoring model using the cut-off values.

Performance metrics	Development cohort		External validation cohort	
	Low risk	High risk	Low risk	High risk
Total, N (%)	23,508 (67.9)	11,134 (32.1)	7537 (69.1)	3365 (30.9)
Predicted score (mean, range)	(2.2, 0–6)	(13.8, 7–35)	(2.2, 0–6)	(13.9, 7–31)
Actual ED visit, N (% of subgroup)	843 (3.6)	1958 (17.6)	291 (3.9)	590 (17.5)
Sensitivity, % (95% CI)	69.9 (68.2–71.6)		67.0 (63.9–70.1)	
Specificity, % (95% CI)	71.2 (70.7–71.7)		72.3 (71.4–73.2)	

*ED* emergency department.

### Post-hoc subpopulation sensitivity analysis

The broad range of applicability and performance consistency of the developed risk-scoring models was determined by conducting a sensitivity analysis across subpopulations (Table 4). Notably, the model performance was found to be the highest in patients with breast cancer and the lowest in patients with colon cancer. However, the performance did not differ significantly among the different types of anti-neoplastic agents.

**Table 4:** Performance of the risk-scoring model among different subpopulations.

Subpopulation	AUROC (95% CI)
Prostate cancer	0.72 (0.67–0.76)
Breast cancer	0.76 (0.71–0.82)
Lung cancer	0.74 (0.71–0.78)
Colon cancer	0.66 (0.60–0.72)
Endocrine agent only	0.71 (0.66–0.75)
Targeted agent-based	0.73 (0.67–0.79)
Cytotoxic agent-based	0.70 (0.67–0.72)

*AUROC* area under the receiver operating characteristic curve, *CI* confidence interval.

## Discussion

We developed a claims-based risk-scoring model focusing on medication variables as a screening tool to classify older patients receiving anti-neoplastic therapy who are at high risk of ED visits. The characteristics included in the risk score are easily accessible to clinicians or pharmacists and can be detected in medical records or prescription data, facilitating the calculation of personalized risk estimates for ED visits.

In the external validation cohort, our model demonstrated acceptable and moderately strong prediction performance, with a C-statistic of 0.76. This performance is notably better than those reported in previous studies^10,11,15,16^. For instance, Brooks GA et al. developed two models with C-statistics of 0.71 and 0.69, respectively^10,11^, and Sutradhar et al.'s model achieved a C-statistic of 0.737^16^. Moreover, Grant et al.^15^ developed a logistic regression model to predict acute care use in cancer patients initiating systemic cancer therapy, which showed a C-statistic of 0.61 in a population-based cohort of 12,162 patients. Their model included three variables: a combination of cancer type and treatment regimen, age, and ED visits in the previous year. Our study, while not focusing on individual chemotherapy regimens, successfully developed a distinct predictive model for ED visits in older patients receiving anti-neoplastic therapy, placing special emphasis on modifiable variables like medication use.

In this study, the incidence of ED visits was relatively low, accounting for only 8.1% of the study population. This finding is in contrast with those of previous studies (ranging from 14.5 to 61.0%)^15,18,19^. These differences could be attributed to the outcome definitions, patient populations, clinical settings, and databases used for analysis.

The strongest predictor of ED visits during anti-neoplastic therapy was prior ED visits within 3 months prior to anti-neoplastic therapy. This finding aligns with previous studies investigating ED visit prediction models for patients with cancer, where prior ED visits were identified as significant predictors^15,20–22^. Our results suggest that patients with a history of ED visits may require additional precautions to prevent future ED visits.

In comorbidities, atrial fibrillation and major bleeding were included in the final model, consistent with previous studies showing that these were the main symptoms of ED visits in older patients or patients with cancer^23,24^.

Among the final predictors, medication variables encompassed general PIM (megestrol), geriatric DDIs (use of three or more CNS-active drugs, regular use of opioids without laxatives), chemotherapeutic DDIs, and the use of 10 or more medications, which is in line with our previous study^5^ that demonstrated that one or more PIMs, geriatric DDIs, chemotherapeutic DDIs, and 10 or more medications increased the risk of ED visits in older patients receiving anti-neoplastic therapy, although individual PIMs were not analyzed. Furthermore, lung cancer and anti-neoplastic therapy with cytotoxic agents, included as final predictors, were more likely to indicate inappropriate polypharmacy in older adults receiving anti-neoplastic therapy^5^.

To the best of our knowledge, this study is the first to investigate risk predictors, including medication variables that may lead to ED visits, among older patients receiving anti-neoplastic therapy, including solid and hematologic malignant neoplasms, using data derived from a national claims database. Our study has several implications for clinical practice and policies. The risk-scoring model developed in our study can be used as a screening tool to identify high-risk older patients receiving anti-neoplastic therapy who are prone to ED visits. This enables the early identification of high-risk patients and targeted interventions, such as medication review and counseling, to prevent ED visits and improve patient outcomes. Furthermore, healthcare policymakers can utilize this model to develop interventions and allocate resources effectively to reduce the risk of ED visits. This could include targeted outreach and education programs for patients and healthcare providers and the development of specialized clinics or telemedicine services to provide timely and appropriate care to patients at high risk of ED visits. While our study establishes a foundation for a novel predictive model, we recognize potential barriers in integrating this model into existing healthcare systems. These may include technical challenges, as well as resistance from policymakers and healthcare professionals. Additionally, conducting a cost-effectiveness analysis is crucial to determine the feasibility and economic viability of implementing this model in clinical settings. Addressing these aspects is essential for the successful adoption and practical application of the model.

This study has several limitations. First, our model tends to overestimate the likelihood of ED visits, particularly at higher risk scores, which could be interpreted as an indicator for increased support and intervention. Therefore, while our model may tend to overestimate, this characteristic could be deemed acceptable when the model is used in tandem with clinical assessments. Second, the model's sensitivity of 0.67 suggest a potential misclassification rate of approximately 33% among those classified as high-risk, coupled with wide confidence intervals, indicating variability in performance that must be considered in clinical application. Third, the limitations inherent in the claims data precluded the consideration of factors such as social determinants of health, specific laboratory results, and medications available without a prescription, all crucial for understanding patient health outcomes, particularly in older populations that often have complex health needs. Fourth, our study did not account for the individual anti-neoplastic therapy regimens and their intensity due to limitations in obtaining accurate dosage and treatment schedule from claims data. Fifth, the external validation’s effectiveness might be overestimated since it used the same administrative data source as our development cohort. Further validation in diverse real-world settings is necessary to confirm the model’s robustness. Sixth, it's crucial to note that our study design and analysis are primarily geared towards predictive modeling, not to establish causation. This distinction is important for interpreting and applying our results in a clinical setting, as the identified associations do not necessarily imply causal relationships Lastly, our study, constrained by the nature of claims data, didn’t differentiate between avoidable and unavoidable ED visits, focusing instead on overall acute care utilization. This limits our ability to identify preventable acute care uses, underlining the need for future research in this area.

In conclusion, our study developed and validated a risk-score model for predicting ED visits among older patients receiving anti-neoplastic therapy, demonstrating moderate discrimination capabilities. While this model provides valuable insights and hold potential in identifying high-risk patients, it necessitates further validation to confirm its effectiveness across diverse healthcare settings and to comprehensively evaluate its impact on patient care.

## Methods

### Study design and database source

We conducted a retrospective cohort study utilizing the annual National Adult Patient Sample (APS) database sourced from the Korean Health Insurance Review and Assessment Service (HIRA) spanning the years 2016 to 2019. This dataset comprises 4,766,420 patients aged 65 years or older, representing 20% of the elderly population before 2016 and reducing to 10% after 2017. The HIRA-APS compilation, conducted annually, involved anonymizing personal information and utilizing stratified sampling. This probabilistic approach of sample extraction, based on sex and age, was employed to ensure data representativeness^25^.

Within HIRA-APS, comprehensive data is available on demographic characteristics, healthcare utilization, prescriptions, and diagnoses. Notably, it provides detailed drug information covering both inpatient and outpatient prescriptions for all patients involved. This extensive data availability was a pivotal factor in selecting a retrospective cohort design for our study. This approach enabled us to utilize a large volume of existing data, crucial for evaluating the impact of medication-related factors on ED visits over multiple years. The retrospective design also helped reduce selection bias and improve generalizability by including a diverse patient population from a national database. Additionally, it provided an efficient way to analyze historical data, avoiding the complexities and time constraints associated with prospective data collection. This method aligns well with our goal to assess a wide array of medication-related factors in older patients on anti-neoplastic therapy and their impact on health outcomes.

This study was conducted in accordance with the transparent reporting of a multivariable prediction model for individual prognosis or diagnosis (TRIPOD) statement^26^. This study was approved by the Seoul National University Institutional Review Board (No. E2002/001-008). The informed consent from the participants was waived by the Institutional Review Board because this study used de-identified data. All methods were performed according to relevant guidelines and regulations.

### Study population and outcome definition

To identify the study population, we selected patients who were prescribed anti-neoplastic agents and also had diagnostic codes for cancer. The anti-neoplastic agents were identified using their Anatomical Therapeutic Chemical (ATC) codes and classified as cytotoxic agent (ATC code L01, except for targeted therapy), targeted agent (ATC codes L01XC, L01XE), and endocrine agent (ATC code L02). The diagnostic codes for cancer were based on the International Statistical Classification of Diseases and Related Health Problems, 10th Revision (ICD10), which included codes C00-C96 (cancer) and D37-D48 (neoplasms of uncertain or unknown behavior, polycythemia vera, and myelodysplastic syndromes).

Given that the HIRA-APS database is an annual database, we included only those received anti-neoplastic therapy after July each year. This timing was selected to establish a baseline period that allow for confirmation of comorbidities, healthcare utilization pattern, and history of anti-neoplastic therapy. We also applied exclusion criteria for patients hospitalized for more than 150 days from January to June, as prolonged hospitalization during this period could significantly affect our outcome. Similarly, patients hospitalized for more than 30 days from their entry date were excluded because the outcome could not occur. The development cohort was constructed from the 2016–2018 APS database, whereas the external validation cohort was constructed from the 2019 APS (Fig. 1). The entry date was defined as the first date on which the anti-neoplastic agent was prescribed after July. The outcome was an initial visit to the ED within 30 days of entry date (Fig. S2).

### Predictor variables

Predictor variables were selected based on factors previously identified as being associated with ED visits in a national claims data study^5^. This study assessed variables highlighted in earlier literature and the lists of potentially inappropriate medications for the elderly, as outlined in established guidelines^27,28^. The collected data included (1) demographic information such as age, sex, and insurance type; (2) ED visit history; (3) Charlson comorbidity index score; (4) presence or history of comorbid conditions such as anemia, congestive heart failure, diabetes, major bleeding, and hypertension (Table S4); (5) cancer diagnosis; and (6) medication variables including the type of anti-neoplastic agents, initiation of anti-neoplastic therapy, number of chronic medications, and general and disease-specific PIMs, and DDIs. General and disease-specific PIMs prescribed during anti-neoplastic therapy were evaluated based on the 2019 American Geriatrics Society Beers Criteria^®^^27^ and screening tool of older people’s prescriptions (STOPP) criteria^28^ for inappropriate use in the geriatric population. We screened for clinically significant DDIs, categorizing them as geriatric and chemotherapeutic DDIs. We identified geriatric DDIs, which are drug interactions that should be avoided in older adults according to the 2019 American Geriatrics Society Beers Criteria^®^ and STOPP criteria, and chemotherapeutic DDIs, which are potentially significant interactions involving anti-neoplastic agents according to a reference database. For chemotherapeutic DDIs, those categorized as “D” or “X” by Lexicomp Online™ or those categorized as “major” or “contraindications” in severity by Micromedex^®^ were considered clinically significant (Table S5). Medication exposure was assessed based on medications used for more than three days during the one week before the entry date (Fig. S2).

Since we utilized a claims database, it was assumed that the absence of a record corresponded to the absence of the corresponding condition. No missing data was observed for demographic factors such as age group, sex, and insurance type.

### Risk-scoring model development, validation and statistical analysis

We summarized the baseline characteristics of the study population using descriptive statistics. To facilitate the integration into a risk-scoring model, we transformed continuous variables into categorical variables. In the training cohort, we developed an ED visit risk-scoring model using LASSO regression method. The choice of LASSO was driven by its efficiency in handling high-dimensional data, a feature of our dataset, and its capability to minimize variable collinearity^29,30^. Unlike methods using L2 regularization (e.g., Ridge regression), LASSO employs L1 regularization, penalizing less significant variables by shrinking their coefficient to zero^30^. This feature selection effectively simplifies the model and reduces overfitting risk. With a large number of potential predictors in our dataset, LASSO's dual function of variable selection and regularization was particularly advantageous, enhancing the model's predictive accuracy and interpretability by focusing on the most significant variables^29,30^. After labeling outcome parameters and predictor variables generation, the risk-scoring model building process was as follows. First, the frequencies of variables were evaluated, and those with a prevalence of less than 1% were excluded. Second, we assessed multicollinearity between the variables using the variance inflation factor. Third, we performed LASSO regression on the training dataset to select predictor variables and to fit the model. The optimal penalty parameter λ for maximizing model performance was determined using tenfold cross-validation. Furthermore, we eliminated variables from the final list if their regression coefficient values, multiplied by 100, were below 1 to enhance the usability of the prediction scores. Subsequently, we obtain the regression coefficients for each variable from the final LASSO selection operator regressions. A risk-score model was developed by assigning a risk score to each variable, multiplying its β coefficient by 100, and rounding it to the nearest integer. Individual risk was determined by summing the weighted scores associated with each assigned risk factor score.

In the external validation cohort, we assessed the model performance in terms of discrimination and calibration^31^. Discrimination was assessed using AUROC. Calibration for comparing the predicted and observed risks was performed using the calibration plot. We categorized patients into low- and high-risk groups for clinical decision-making based on their risk score distribution and predicted probability in the development cohort. The Youden index was used to determine the cut-off point for the high-risk group, which balances sensitivity and specificity^32^. Sensitivity and specificity were evaluated for these cut-off values.

SAS version 9.4 (SAS Institute, Cary, NC, USA) was used for the data management and descriptive statistics. LASSO regression was performed using the ‘glmnet’ package in the R statistical software (R Foundation for Statistical Computing, Vienna, Austria).

### Post-hoc subpopulation sensitivity analysis

The performance of the risk-scoring model was assessed in subgroups of patients with diverse cancer diagnoses and those who were treated with different types of anti-neoplastic agents: cytotoxic agent-based, targeted agent-based, and endocrine agent-only.

### Supplementary Information

Supplementary Information.

## Data Availability

The dataset supporting the conclusions of this article is available from the Korea National Health Insurance Service (KNHIS) Data Sharing Service homepage (https://nhiss.nhis.or.kr/bd/ab/bdaba001cv.do) but restrictions apply to the availability of these data. The KNHIS, the data provider, requires all involved researchers to pledge not to share, release, or review the data with other entities.
