# Supplementary material for: Development of a claims-based risk-scoring model to predict emergency department visits in older patients receiving anti-neoplastic therapy
Source: Sci Rep. 2024 Jan 17;14:1485. doi: 10.1038/s41598-024-51981-0 (PMC10794170; doi:10.1038/s41598-024-51981-0)

SUPPLEMENTARY MATERIAL

Development of a claims-based risk-scoring model to predict emergency department visits in older patients receiving anti-neoplastic therapy

Yewon Suh[1], Jonghyun Jeong[2], Soh Mee Park[1], Kyu-Nam Heo[2], Mee Yeon Lee[2], Young-Mi Ah[3], Jin Won Kim[4], Kwang-Il Kim[5], Ju-Yeun Lee[1]

Table S1. The list of variables considered in the analyses.

Table S2. Predicted probability of emergency department visits by the risk scores and observed percentages in development and external validation cohorts.

Table S3. The three cases as high-risk patients from external validation dataset.

Table S4. Diagnosis and ICD Codes used in the study.

Table S5. The list of chemotherapeutic drug-drug interactions.

Fig S1. Selection of parameters at varying log-transformed lambda values. (A) Tuning parameter selection in the LASSO model used 10-fold cross-validation. (B) LASSO coefficient profile plot of the 50 features.

Fig S2. Diagram of the design.

Table S1. The list of variables considered in the analyses.

No	Group of variables	Variables
1	Demographic	Age group
2		Insurance type
3		Sex
4		Receiving antineoplastic therapy in an outpatient setting
5	ED visit experience	Prior ED visits within 3 months
6	Cancer diagnosis	Breast cancer
7		Colorectal cancer
8		Esophagus cancer
9		Gastric cancer
10		Head and neck cancer
11		Leukemia
12		Lung cancer
13		Lymphoma
14		Prostate cancer
15		Renal cancer
16	Charlson comorbidity index score	Charlson comorbidity index score group
17	Comorbidities or prior condition	Anemia
18		Atrial fibrillation
19		Cerebrovascular disease
20		Chronic obstructive pulmonary disease
21		Congestive heart failure
22		Dementia
24		Diabetes Mellitus
24		Hypertension
25		Liver disease (moderate to severe)
26		Major bleeding
27		Myocardial infarction
28		Peptic ulcer disease
29		Peripheral vascular disease
30		Renal disease (severe)
31		Rheumatic disease
32	Disease specific potentially inappropriate medication (PIM)_Beers criteria	Chronic kidney disease- traditional nonsteroidal anti-inflammatory drugs
33		Delirium-Antipsychotics
34		Delirium-Benzodiazepines
35		Delirium-Corticosteroids
36		Delirium-H2 receptor antagonist
37		Delirium-Strong anticholinergic
38		Delirium-Nonbenzodiazepine benzodiazepine receptor agonist hypnotics
39		Dementia-Antipsychotics
40		Dementia-Benzodiazepines
41		Dementia-Strong anticholinergic
42		Dementia- Nonbenzodiazepine benzodiazepine receptor agonist hypnotics
43		Fall or fracture-CNS active drugs
44		Gastric or duodenal ulcer-Traditional nonsteroidal anti-inflammatory drugs including aspirin high dose without proton pump inhibitor
45		Heart Failure-Cilostazole
46		Heart Failure-Dronedarone
47		Heart Failure-lobeglitazone, pioglitazone
48		Heart Failure-Nondihydropyridine calcium channel blockers
49		Heart Failure-Nonsteroidal anti-inflammatory drugs

50		Lower urinary tract symptoms, benign prostatic hyperplasia-Strong anticholinergic except for urinary antimuscarinics
51		Parkinson's disease-Antipsychotics except for clozapine, quetiapine
52		Parkinson's disease-Metoclopramide
53		Syncope-Acetylcholinesterase inhibitors
54		Urinary incontinence in Women-Alpha1 blockers
55		Urinary incontinence in Women-Estrogens
56	Disease specific PIM_ The Screening Tool of Older Persons' potentially inappropriate Prescriptions (STOPP) criteria	Atrial fibrillation without stent-Anticoagulants, antiplatelets
57		Bradycardia-Acetylcholinesterase inhibitors
58		Bradycardia-Beta blockers
59		Constipation without laxatives-Strong anticholinergics, opioids, tramadol, verapamil
60		Gout-Thiazide diuretics
61		Hyperkalemia-Renin-angiotensin system inhibitors or potassium-sparing diuretics
62		Narrow angle glaucoma-Strong anticholinergics
63	Drug-Drug interation PIM_Beers criteria	2 or more anticholinergic drug
64		3 or more CNS active drug
65		2 or more renin-angiotensin system inhibitors or potassium-sparing diuretics
66		Alpha1 blockers-Loop diuretics
67		Lithium- Renin-angiotensin system inhibitors
68		Lithium-Loop diuretics
69		Opioids-Benzodiazepines
70		Opioids-Gabapentin/Pregabalin
71		Phenytoin-sulfamethoxazole and trimethoprim
72		Theophylline-Cimetidine
73		Theophylline-Ciprofloxacin
74		Warfarin- Traditional nonsteroidal anti-inflammatory drugs
75		Warfarin-Amiodarone
76		Warfarin-Ciprofloxacin
77		Warfarin-Macrolides except for azithromycin
78		Warfarin-sulfamethoxazole and trimethoprim
79	Drug-Drug interation PIM_STOPP criteria	Beta blockers- Nondihydropyridine calcium channel blockers
80		Donepezil, rivastigmine, galantamine-Beta blockers, Digoxin, Nondihydropyridine calcium channel blockers
81		Longterm steroid without Vitamin D, Ca, Antiosteoporotic agents
82		Methotrexate for RA without folic acid
83		Regular opioid without laxatives
84	General PIM_Beers criteria	Antispasmodics
85		1st generation Antihistamines
86		Amiodarone
87		Anticholinergic antipsychotics
88		Anticholinergic muscle relaxants
89		Antiparkinsonian agents, anticholinergic
90		Atypical antipsychotics
91		Barbiturate
92		Desmopressin
93		Digoxin
94		Estrogen, Testosterone
95		Glibenclamide, Glimepiride
96		Growth hormone
97		Ketorolac
98		Benzodiazepines
99		Megestrol
100		Metoclopramide

101		Nifedipine, immediate release
102		Nitrofurantoin
103		Non selective alpha-1 blockers
104		Proton pump inhibitor
105		Skeletal muscle relaxants
106		Ticlopidine
107		Traditional nonsteroidal anti-inflammatory drugs (excluded ketorolac)
108		Tricyclic antidepressant
109		Typical antipsychotics
110		Urinary antimuscarinics
111		Nonbenzodiazepine benzodiazepine receptor agonist hypnotics
112	General PIM_STOPP criteria	Multiple Antiplatelets
113		Multiple selective serotonin reuptake inhibitors
114		Multiple renin-angiotensin system inhibitors
115		Multiple Anticoagulants
116	Medication-related variables	Newly started anti-neoplastic therapy
117		Number of chronic medications group
118		Type of antineoplastic agents
119		Anticholinergic burden group
120		Chemotherapeutic drug-drug interactions

Table S2. Predicted probability of emergency department visits by the risk scores and observed percentages in development and external validation cohorts.

Score	Probability	Patients, No./Total No. (%)	
		Development cohort	External validation cohort
0	0.032	149/7705 (1.9)	44/2555 (1.7)
1	0.036	63/2668 (2.4)	21/764 (2.7)
5	0.056	135/2290 (5.9)	49/788 (6.2)
6	0.062	170/2135 (8.0)	44/640 (6.9)
7	0.070	135/1473 (9.2)	44/466 (9.4)
8	0.078	149/1239 (12.0)	26/341 (7.6)
9	0.086	155/1092 (14.2)	33/323 (10.2)
10	0.096	105/678 (15.5)	29/194 (14.9)
11	0.107	82/489 (16.8)	28/136 (20.6)
12	0.119	58/423 (13.7)	19/116 (16.4)
13	0.131	97/603 (16.1)	22/188 (11.7)
14	0.145	72/454 (15.9)	25/152 (16.4)
15	0.161	67/389 (17.2)	25/124 (20.2)
16	0.177	85/491 (17.3)	41/193 (21.2)
17	0.195	96/500 (19.2)	31/146 (21.2)
18	0.214	114/551 (20.7)	38/185 (20.5)
19	0.234	120/552 (21.7)	41/164 (25.0)
20	0.256	109/487 (22.4)	31/131 (23.7)
21	0.279	97/386 (25.1)	34/116 (29.3)
22	0.303	109/344 (31.7)	41/119 (34.5)
23	0.328	85/289 (29.4)	19/73 (26.0)
24	0.355	73/213 (34.3)	23/64 (35.9)
25	0.382	42/166 (25.3)	16/45 (35.6)
26	0.410	41/120 (34.2)	7/34 (20.6)
27	0.439	28/81 (34.6)	2/13 (15.4)
28	0.468	20/59 (33.9)	11/26 (42.3)
29	0.497	11/26 (42.3)	3/6 (50.0)
32	0.584	0/2 (0)	0/0 (0)
35	0.666	1/1 (100)	0/0 (0)

Table S3. The three cases predicted as high-risk patients from external validation dataset.

Case	Risk score	ED visit
Prior ED visits within 3 months, Lung cancer, Major bleeding, Newly started anti-neoplastic therapy, Cytotoxic agents-based anti-neoplastic therapy, Chemotherapeutic drug-drug interactions, Three or more CNS-active drugs, Regular opioids without laxatives, 10 or more medications<br>(Medication: acetylcysteine, almagate, aprepitant, butylscopolamine, chlorpromazine, dexamethasone, fentanyl, gabapentin, metoclopramide, palonosetron, pantoprazole, capecitabine, cisplatin, trastuzumab)	31	No
Prior ED visits within 3 months, Lung cancer, 6 or more CCI score, Newly started anti-neoplastic therapy, Cytotoxic agents-based anti-neoplastic therapy, Three or more CNS-active drugs, Regular opioids without laxatives, Megestrol, 10 or more medications<br>(Medication: folic acid, chlorpheniramine, cobamamide, epinephrine, fentanyl, lidocaine, midazolam, oxycodone and naloxone, megestrol, metoclopramide, pemetrexed)	31	Yes
Prior ED visits within 3 months, Lung cancer, Major bleeding, 6 or more CCI score, Newly started anti-neoplastic therapy, Cytotoxic agents-based anti-neoplastic therapy, Three or more CNS-active drugs, Megestrol, 10 or more medications<br>(Medication: acetylcysteine, alprazolam, chlorpheniramine, clopidogrel, codeine, furosemide, glimepiride, levodropropizine, megestrol, metformin, methylephedrine, metoclopramide, midazolam, omeprazole, palonosetron, piperacillin and tazobactam, rosuvastatin, Theophylline, tranexamic acid, carboplatin, etoposide)	29	Yes

Table S4. Diagnosis and ICD Codes used in the study.

Diagnosis	ICD-10-CM code
Anemia	D461, D464, D50-D64
Atrial fibrillation	I48
Cerebrovascular disease	G45, I63, I64
Chronic obstructive pulmonary disease	J40-J47, J60-J67, J684, J701, J703
Congestive Heart failure	I109-I110, I130, I132, I255, I42, I43, I50
Dementia	F00-F03,F051, G30, G311
Diabetes mellitus	E10, E11, E12, E13, E14, E102-E105, E107, E112-E114, E117, E122-E125, E127, E132-E135, E137, E142-E145, E147
Hypertension	I10-I13, I15
Liver disease(moderate to severe)	I850, I859, I864, K704, K711, K721, K729, K76
Major bleeding	D62, I60-I62, I690, I691, I692, J942, K250, K252, K254, K256, K260, K262, K264, K266, K270, K272, K280, K282, K284, K286, K920, K921, K922, N02, R04, R31
Myocardial infarction	I21-I22, I252
Peptic ulcer disease	K25-K28
Peripheral vascular disease	I70-I71, I731, I738-I739, I771, I790-I791, I798, K551, K558, K559, Z958-Z959
Renal disease(severe)	I120, I132, N185, N19, N250, Z49, Z992
Rheumatologic disease	M05, M06, M315, M32-M34, M351, M353, M360

Table S5. The list of chemotherapeutic drug-drug interactions.

Antineoplastic agents	Interacting Drug and Class
**Cytotoxic agent**	
arsenic trioxide	levofloxacin, voriconazole
asparaginase	prednisolone
bleomycin	leflunomide
bortezomib	ascorbic acid, carbamazepine, rifampicin
busulfan	metronidazole
capecitabine	allopurinol, cimetidine, fosphenytoin, leflunomide, metronidazole, proton pump inhibitors, warfarin
carboplatin	candesartan, leflunomide, roflumilast, warfarin
cisplatin	furosemide, leflunomide, roflumilast, thioctic acid, valproic acid
cyclophosphamide	carbamazepine, cyclosporine, hydrochlorothiazide, phenytoin, warfarin
docetaxel	amiodarone, clarithromycin, CYP3A4 inducers(strong), itraconazole, roflumilast
doxorubicin	amiodarone, amitriptyline, aprepitant, azoles, bupropion, carvedilol, chlorpromazine, ciprofloxacin, clobazam, cyclosporine, CYP3A4 inducers(strong), dexamethasone, dexrazoxane, diltiazem, dronedarone, duloxetine, fluoxetine, fluvoxamine, leflunomide, macrolides, mirabegron, morphine, paroxetine, propafenone, roflumilast, terbinafine, ticagrelor, verapamil, warfarin
epirubicin	cimetidine
eribulin	fluconazole, hydroxyzine
etoposide	cyclosporine, CYP3A4 inducers(strong), roflumilast, warfarin
fluorouracil	allopurinol, cimetidine, metronidazole, roflumilast, warfarin
gemcitabine	leflunomide, warfarin
hydroxyurea	leflunomide
ifosfamide	azoles, ciprofloxacin, clarithromycin, diltiazem, pioglitazone
irinotecan	azoles, carbamazepine, clarithromycin, rifampicin, roflumilast
mercaptopurine	allopurinol, azathioprine, mesalazine, mycophenolic acid, perindopril, sulfamethoxazole/trimethoprim, sulfasalazine, warfarin
methotrexate	acetylsalicylic acid, acitretin, amoxicillin, ampicillin, chloral hydrate, cyclosporine, doxycycline, fosphenytoin, furosemide, hydrochlorothiazide, levetiracetam, NSAIDs, phenytoin, piperacillin, proton pump inhibitors, roflumilast, torasemide. warfarin
paclitaxel	amiodarone, azoles, candesartan, clarithromycin, clopidogrel, CYP3A4 inducers(strong), estradiol, oxcarbazepine
pemetrexed	NSAIDs, tolvaptan
tegafur, combinations	phenytoin, warfarin
temsirolimus	fluconazole
topotecan	carvedilol, clopidogrel, tolvaptan
tretinoin	multivitamins, tranexamic acid
vinblastine	azoles, leflunomide, phenytoin, rifampicin

vincristine	amiodarone, azoles, carbamazepine, carvedilol, cyclosporine, diltiazem, dronedarone, felodipine, leflunomide, macrolides, phenytoin, ticagrelor, warfarin
vinorelbine	clarithromycin, gefitinib, itraconazole
**Targeted agent**	
afatinib	amiodarone, carvedilol, dronedarone, macrolides, phenobarbital, rifampicin, verapamil
ceritinib	aceclofenac, amlodipine, atorvastatin, celecoxib, ciprofloxacin, clarithromycin, dexamethasone, diclofenac, domperidone, fentanyl, granisetron, hydrocortisone, itraconazole, levofloxacin, naproxen, olanzapine, oxycodone, tramadol, zolpidem
crizotinib	alfentanil, amiodarone, amitriptyline, aprepitant, carvedilol, cilostazol, codeine, dihydrocodeine, diltiazem, domperidone, donepezil, escitalopram, fentanyl, fluconazole, haloperidol, hydrocodone, hydroxyzine, imipramine, macrolides, metronidazole, nortriptyline, oxycodone, perphenazine, propranolol, quetiapine, quinolones, rifampicin, risperidone, solifenacin, tizanidine, tolterodine, triazolam, triptorelin, venlafaxine,
dasatinib	amiodarone, amitriptyline, antacids(Al, Ca, Mg), azoles, buprenorphine, carbamazepine, clarithromycin, domperidone, donepezil, ebastine, escitalopram, formoterol, granisetron, H2 blocker, hydroxyzine, imipramine, leflunomide, paracetamol, perphenazine, proton pump inhibitors, quetiapine, quinolones, solifenacin, tacrolimus, trazodone
erlotinib	antacids(Al, Ca, Mg), ciprofloxacin, clarithromycin, H2 blocker, itraconazole, proton pump inhibitors, rifampicin, warfarin
everolimus	aprepitant, azoles, carbamazepine, clarithromycin, diltiazem, dronedarone, leflunomide, verapamil
gefitinib	antacids(Al, Ca, Mg), bupropion, CYP3A4 inducers (strong), fluoxetine, H2 blocker, hydrotalcite, propafenone, proton pump inhibitors
ibrutinib	acetylsalicylic acid, azoles, cilostazol, ciprofloxacin, clopidogrel, enoxaparin, heparin, rifampicin, ticlopidine
imatinib	amiodarone, aprepitant, buprenorphine, cilostazol, codeine, colchicine, CYP3A4 inducers(strong), dexamethasone, dihydrocodeine, domperidone, ergotamine, fentanyl, ibuprofen, ivabradine, oxycodone, paracetamol, pethidine, tacrolimus, tramadol, warfarin
lapatinib	aripiprazole, clarithromycin, dexamethasone, domperidone, donepezil, escitalopram, fluconazole, fluoxetine, levosulpride, nortriptyline, quetiapine, quinolones, sertraline, trazodone
nilotinib	amiodarone, amitriptyline, antacids(Al, Ca, Mg), azoles, cilostazol, codeine, dexamethasone, dihydrocodeine, diltiazem, domperidone, donepezil, ebastine, escitalopram, fentanyl, formoterol, H2 blocker, haloperidol, hydroxyzine, ivabradine, levosulpride, macrolides, metronidazole, midazolam, morphine, oxycodone, proton pump inhibitors, quetiapine, quinolones, rifampicin, tacrolimus, ticagrelor, trazodone, warfarin
palbociclib	atorvastatin, codeine, fentanyl, itraconazole, oxycodone, tramadol

pazopanib	amiodarone, antacids(Al, Ca, Mg), atorvastatin, azoles, buprenorphine, carbamazepine, carvedilol, chlorpromazine, domperidone, dronedarone, escitalopram, fentanyl, granisetron, H2 blocker, haloperidol, hydrotalcite, hydroxyzine, macrolides, nortriptyline, olanzapine, ondansetron, propafenone, proton pump inhibitors, quetiapine, quinolones, solifenacin, tizanidine, tolterodine, trazodone, verapamil
radotinib	clarithromycin
regorafenib	clarithromycin
ruxolitinib	azoles, clarithromycin
sorafenib	alfuzosin, amiodarone, amitriptyline, amoxapine, azoles, buprenorphine, domperidone, donepezil, escitalopram, fluoxetine, granisetron, haloperidol, hydroxyzine, imipramine, macrolides, metronidazole, nortriptyline, ondansetron, paracetamol, prednisolone, propacetamol, quetiapine, quinolones, rifampicin, solifenacin, tacrolimus, trazodone, warfarin
sunitinib	alfuzosin, amiodarone, amitriptyline, azoles, buprenorphine, dexamethasone, domperidone, donepezil, escitalopram, haloperidol, hydroxyzine, ivabradine, macrolides, metronidazole, morphine, nortriptyline, ondansetron, proton pump inhibitors, quetiapine, quinolones, trazodone
vandetanib	hydroxyzine
**Endocrine agent**	
abiraterone	doxorubicin, mequitazine, selexipag
anastrozole	estrogen conjugated
bicalutamide	domperidone, warfarin
degarelix	alfuzosin, amitriptyline, buprenorphine, donepezil, escitalopram, famotidine, imipramine, metronidazole, quetiapine, quinolones, risperidone
enzalutamide	alfentanil, amiodarone, buprenorphine, carbamazepine, celecoxib, clarithromycin, codeine, dexamethasone, diazepam, dihydrocodeine, escitalopram, fentanyl, gemigliptin, glimepiride, itraconazole, ivabradine, linagliptin, losartan, meloxicam, methylprednisolone, nifedipine, ondansetron, oxycodone, proton pump inhibitors, quetiapine, rivaroxaban, sulfamethoxazole/trimethoprim, tacrolimus, torasemide, tramadol, triazolam, warfarin
estramustine	calcium, donepezil
exemestane	carbamazepine, estradiol
goserelin	alfuzosin, amiodarone, amitriptyline, aripiprazole, azithromycin, azoles, buprenorphine, domperidone, donepezil, dronedarone, escitalopram, flecainide, fluoxetine, haloperidol, hydroxyzine, metronidazole, olanzapine, paroxetine, perphenazine, pimozide, propafenone, quetiapine, quinolones, risperidone, sertraline, sulpiride, tizanidine, tolterodine, trazodone, venlafaxine
letrozole	cilostazol, clarithromycin

leuprorelin	alfuzosin, amiodarone, amitriptyline, aripiprazole, azithromycin, azoles, bedaquiline, buprenorphine, citalopram, clarithromycin, clomipramine, clozapine, domperidone, donepezil, doxepin, dronedarone, ebastine, escitalopram, famotidine, flecainide, fluoxetine, granisetron, haloperidol, hydroxychloroquine, hydroxyzine, imipramine, levosulpride, metronidazole, nortriptyline, olanzapine, ondansetron, paroxetine, perphenazine, propafenone, quetiapine, quinolones, sertraline, solifenacin, sotalol, sulpiride, tacrolimus, tizanidine, tolterodine, trazodone, venlafaxine
megestrol	dabigatran etexilate, enoxaparin, warfarin
tamoxifen	amiodarone, amisulpride, aripiprazole, atazanavir, azoles, buprenorphine, bupropion, cinacalcet, clarithromycin, clobazam, clozapine, CYP3A4 inducers(strong), domperidone, donepezil, dronedarone, duloxetine, escitalopram, fluoxetine, fluvoxamine, hydroxychloroquine, hydroxyzine, ivabradine, levofloxacin, methotrexate, metronidazole, mirabegron, moxifloxacin, ondansetron, paroxetine, quetiapine, sertraline, St. John's Wort, tacrolimus, terbinafine, warfarin, ziprasidone
toremifene	amitriptyline, azoles, carbamazepine, domperidone, donepezil, escitalopram, flecainide, hydroxyzine, ivabradine, macrolides, metronidazole, nortriptyline, quetiapine, quinolones, sertraline, trazodone, venlafaxine
triptorelin	alfuzosin, amiodarone, amitriptyline, aripiprazole, azoles, buprenorphine, chlorpromazine, clarithromycin, domperidone, donepezil, doxepin, escitalopram, fluoxetine, granisetron, haloperidol, hydroxyzine, imipramine, metronidazole, ondansetron, paroxetine, propafenone, quetiapine, quinolones, risperidone, sertraline, solifenacin, tacrolimus, tizanidine, tolterodine, trazodone, venlafaxine

Fig S1. Selection of parameters at varying log-transformed lambda values. (A) Tuning parameter selection in the LASSO model used 10-fold cross-validation. (B) LASSO coefficient profile plot of the 50 features.

(A)

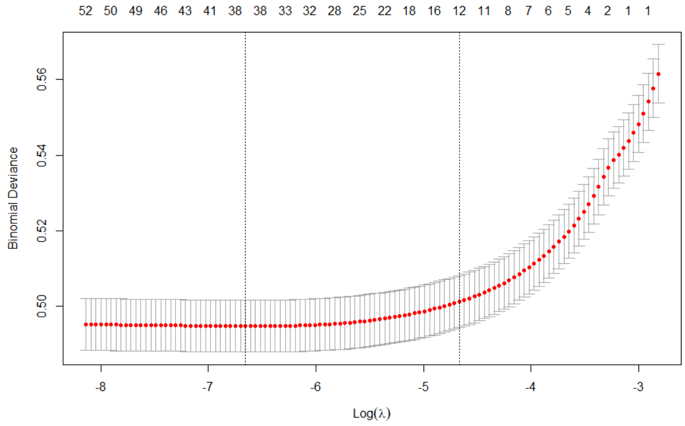

(B)

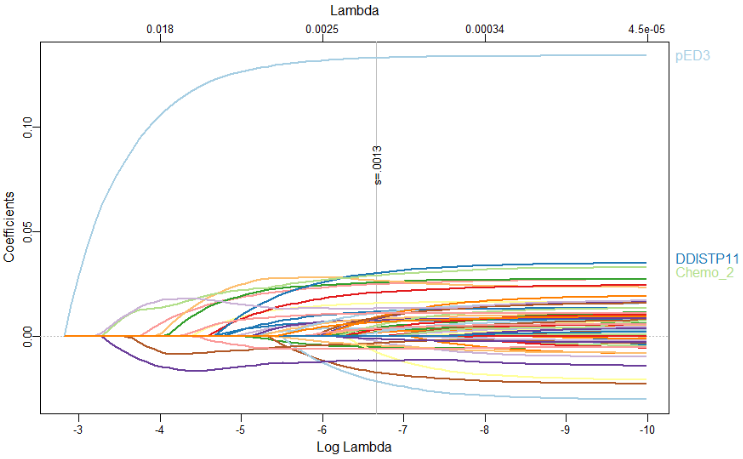

Fig S2. Diagram of the design.

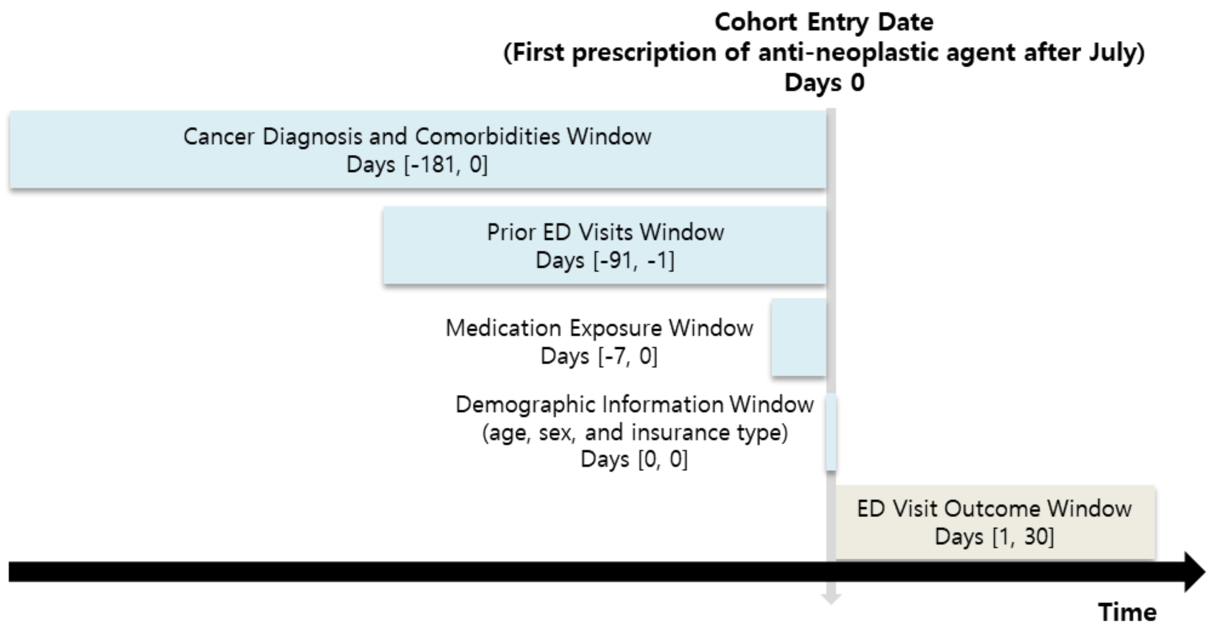

Supplement: Supplementary file 1 — Supplementary Information. [file 41598_2024_51981_MOESM1_ESM.pdf]
